# Oxytocin receptor gene, post-traumatic stress disorder and dissociation in a community sample of European American women

**DOI:** 10.1192/bjo.2022.74

**Published:** 2022-06-03

**Authors:** Hyunhwa Lee, Anthony P. King, Yang Li, Julia S. Seng

**Affiliations:** School of Nursing, University of Nevada, Las Vegas, Nevada, USA; Department of Psychiatry, Ohio State University Wexner Medical Center, Columbus, Ohio, USA; School of Nursing, University of Texas at Austin, Texas, USA; School of Nursing, University of Michigan, Ann Arbor, Michigan, USA

**Keywords:** Oxytocin receptor, post-traumatic stress, dissociation, gene association, childhood maltreatment

## Abstract

The aims of this study were: (a) to examine associations of oxytocin receptor gene (*OXTR*) single nucleotide polymorphisms (SNPs) with post-traumatic stress disorder (PTSD) and dissociative symptoms and (b) to investigate gene–environment (G × E) interaction with childhood maltreatment. Salivary DNA samples from 228 women of European ancestry were analysed. Two SNPs, rs237895 and rs237897, were associated with dissociative symptoms but not PTSD diagnosis. Another SNP (rs2254298) was associated with dissociation when interacting with history of childhood maltreatment. These results contribute to theorising and evidence suggesting that the oxytocin system and its genetics may be associated with risk for dissociation among European American women, including those with maltreatment history. Replication with larger patient samples, including men and other ancestry groups, is needed.

Post-traumatic stress disorder (PTSD) is a heterogeneous disorder with complex causality, including gene–environment (G × E) interactions.^1^ A dissociative subtype of PTSD has been included in DSM-5.^2^ Identifying mechanisms to better understand the biology of this dissociative subtype could advance treatments.

One plausible biological mechanism of dissociation is dysregulation of the oxytocin system,^3^ which cascade theory posits to be vulnerable to dysregulation in infancy and early childhood via relational trauma^4^ and which is a biological system related to many survival functions affected by trauma.^5^ Oxytocin is associated with caregiving and attachment,^6^ and has parasympathetic roles in recovery from stress.^5,7^ The oxytocin system is one of the three pillars of self-regulation posited by cascade theory which adapts in the context of trauma associated with childhood maltreatment.^4^ Oxytocin makes theoretical sense as a common, cross-cutting element of the PTSD dissociative subtype. A small number of studies suggest that the oxytocin receptor gene (*OXTR*) may be relevant to PTSD and dissociation;^8,9^ one links a single nucleotide polymorphism (SNP) in *OXTR* (rs53576) with an unresolved attachment status and higher depersonalisation and derealisation symptoms,^9^ and another finds increased methylation in the *OXTR* promoter in persons with functional neurological disorders.^8^ However, further study of dissociation and *OXTR* is warranted.

Here we present an exploratory analysis from a community sample of pregnant women of European ancestry to examine the association of *OXTR* polymorphisms with dissociative symptoms and PTSD diagnosis and to further investigate the G × E interaction of *OXTR* SNPs with a history of childhood maltreatment.

## Method

### Design

This was a candidate gene association study of pregnant women, either PTSD-positive or trauma-exposed and PTSD-negative, recruited from a study of the effect of PTSD on perinatal outcomes (US National Institutes of Health, J.S.S. and A.P.K., R01 NR 008767-S2). The authors assert that all procedures contributing to this work comply with the ethical standards of the relevant national and institutional committees on human experimentation and with the Helsinki Declaration of 1975, as revised in 2008. All procedures involving human participants were approved by the University of Michigan Institutional Review Board (HUM00010610). Written informed consent was obtained from all participants. Details of the parent study methods, including psychometrics, are reported elsewhere.^10^

### Sample

This analysis was conducted with salivary DNA from 228 European American women from the PTSD-diagnosed and trauma-exposed but resilient cohorts. Of these, 135 had lifetime PTSD and 93 had been exposed to trauma but did not develop PTSD.

### Data collection

The mid-trimester diagnostic interview used validated, reliable measures.^10^ Lifetime PTSD was determined using the National Women's Study DSM-IV PTSD module. Maltreatment was determined by reports on the Life Stressor Checklist of any emotional abuse or neglect, physical abuse or neglect, and contact or penetrative sexual abuse as the index trauma. Dissociation was assessed using the Dissociative Experiences Scale Taxon version (DES-T); a report of ‘sometimes or more often than sometimes’ on the DES-T items for depersonalisation or derealisation was a proxy for the DSM-5 dissociative subtype. The Oragene kit was used for saliva specimen collection (DNA Genotek Inc., Ottawa, Canada).

### Genotyping

Genomic DNA was extracted using a semi-automated filter system (QuickGene, Kurabo Ltd, Osaka, Japan), quantified using PicoGreen (Invitrogen, Carlsbad, California, USA) and genotyped using a custom 4800 SNP Infinium bead-array microarray (Illumina, San Diego, California, USA), at the University of Michigan Advanced Genomics Core. Our microarray included nine *OXTR* SNPs previously associated with PTSD (rs1042778, rs237885, rs237887, rs237888, rs2254298, rs237889, rs53576, rs237895 and rs237897). We used Haploview to determine *OXTR* SNP linkage disequilibrium structure (www.broadinstitute.org). All *OXTR* SNPs were found to be in Hardy–Weinberg equilibrium (all *P* > 0.05). Multiple testing correction accounting for linkage disequilibrium between SNPs was calculated per Nyholt (α < 0.0089 for a type I error rate at 5% for these nine SNPs).

### Data analysis

To test associations of *OXTR* SNPs with the symptom variables, we conducted quantitative trait gene association tests using the symptom counts for PTSD and dissociation for one-way analysis of variance (ANOVA) with a Bonferroni adjustment for *post hoc* comparisons. Independent samples *t*-tests determined which genotype was associated with the dissociation phenotype. We used univariate ANOVA to examine the variance explained by the SNP in comparison with childhood maltreatment history and then to assess whether the risk accrued additively or via G × E interaction.

## Results

### Sample characteristics

Participants had a mean age of 30.9 years. The majority had post-secondary education and household income larger than $15 000. Among these 228 women with trauma exposure, 93 (40.8%) met lifetime PTSD diagnostic criteria. The mean number of lifetime PTSD symptoms was 5.3 (s.d. = 4.96). Dissociation mean score was 1.0 (s.d. = 2.08). Fifteen (16.1%) of the women with PTSD had the dissociative subtype (supplementary Table 1, available at https://doi.org/10.1192/bjo.2022.74).

### Gene associations with dissociation

Results of genotypic association with dissociation score are in Table 1. For rs237895 and rs237897, major C allele carriers reported higher levels of dissociation.

**Table 1:** Dissociation score stratified by *OXTR* single nucleotide polymorphism (SNP) genotypes (*n* = 228)^a^

	Genotype (*n*)			Dissociation score, mean (s.d.)			Carrier 1	
SNP	1/1	1/2	2/2	1/1	1/2	2/2	*F; P*	*t; P*
rs1042778	G/G (81)	G/T (120)	T/T (27)	1.1 (2.4)	1.0 (2.0)	0.6 (0.9)	0.76; 0.467	2.15; 0.035
rs237885	G/G (62)	G/T (117)	T/T (49)	1.0 (1.9)	0.9 (1.7)	1.4 (3.0)	0.79; 0.457	−1.05; 0.300
rs237887	A/A (67)	A/G (120)	G/G (41)	0.9 (1.5)	0.9 (1.9)	1.5 (3.2)	1.24; 0.296	−1.27; 0.209
rs237888	T/T (200)	T/C (27)	C/C (1)	1.0 (2.1)	0.8 (1.7)	4.0^b^	1.20; 0.303	−1.45; 0.150
rs2254298	G/G (169)	G/A (55)	A/A (4)	0.9 (1.8)^c^	1.0 (1.9)	4.0 (8.0)^c^	0.55; 0.621	−0.76; 0.501
rs237889	C/C (87)	C/T (112)	T/T (29)	1.2 (2.6)	0.8 (1.7)	1.1 (1.8)	0.97; 0.382	−0.27; 0.785
rs53576	G/G (111)	G/A (100)	A/A (17)	1.0 (2.1)	1.0 (2.1)	1.1 (2.2)	0.01; 0.992	−0.11; 0.911
rs237895	C/C (88)	C/T (116)	T/T (24)	1.0 (2.2)	1.1 (2.2)	0.3 (0.6)	9.21; **0.00018**	4.29; **0.000037**
rs237897	C/C (92)	C/T (112)	T/T (24)	1.1 (2.3)	1.1 (2.1)	0.3 (0.6)	9.20; **0.00018**	4.29; **0.000037**

a. Results were not corrected for multiple testing, and instead the adjusted *P*-value of 0.0089 was used at the threshold for significance.

b. The minor allele homozygote (C/C) of rs237888 occurs only once in the sample; thus, the standard deviation was not available.

c. Groups with the same letter represent significant differences as tested by one-way analysis of variance with *post hoc* analysis with Bonferroni correction.

To put the genotype into biopsychosocial context, we conducted univariate ANOVA models to compare variance in dissociation explained by the genotypes with variance explained by maltreatment (supplementary Table 2). The major alleles and genotypes account for less variance (*ε^2^*) than the maltreatment variable. All of these alleles and genotypes appeared to be protective when the individual had a maltreatment history. For dissociation, the major G allele in rs2254298 was protective (*P* < 0.001) when interacting with maltreatment (*ε^2^* = 6%). The CC homozygote in rs237897 also appeared protective for dissociation, although not significant with the *P* < 0.0089 multiple testing correction, especially when having more than 1 type of child maltreatment (*ε^2^* = 3%).

## Discussion

In this small community sample of European American women, we found a potentially biologically meaningful association of *OXTR* SNPs rs237895 and rs237897 with dissociative symptoms, and also of the *OXTR* SNP rs2254298 with dissociation when interacting with maltreatment history. These findings indicate that the oxytocin receptor may be responsive to psychological and/or environmental stressors, a hypothesis also supported by epigenetic studies where DNA methylation in the *OXTR* gene occurred after both acute psychological stress^11^ and early-life adversity.^8^ Our finding that a high level of dissociative symptoms was associated with *OXTR* also lends support to theories that alterations in the oxytocin system might play a role in the phenomenon of dissociation characterised by depersonalisation and derealisation symptoms (e.g. cascade theory^4^ and polyvagal theory^5,12^).

Findings are similar to other studies of gene associations with PTSD.^13^ Our preliminary finding of a gene variant in the oxytocin system is consistent with results on hormone levels observed in previous pilot studies, in which PTSD was associated with low plasma concentration of oxytocin^14^ and the PTSD dissociative subtype was associated with very high levels of oxytocin.^14,15^

There are limitations to this non-clinical exploratory study. Recall bias due to self-reporting of maltreatment may have introduced a source of error variance that would decrease power to detect significant relationships. The exclusively European American and exclusively pregnant sample may affect the generalisability. Replication in clinical populations is needed with large samples that include adequate ancestry and gender representation. Given the oxytocin system's multiple functions relevant to dissociation and early-life trauma, studies that consider oxytocin dysregulation as a cross-cutting biological mechanism involved in childhood maltreatment sequelae are warranted.

## Data Availability

The data that support the findings of this study are available from the corresponding author on reasonable request.

## Supplementary material

For supplementary material accompanying this paper visit http://doi.org/10.1192/bjo.2022.74. click here to view supplementary material
