# Supplementary material for: Oxytocin receptor gene, post-traumatic stress disorder and dissociation in a community sample of European American women
Source: BJPsych Open. 2022 Jun 3;8(4):e104. doi: 10.1192/bjo.2022.74 (PMC9230437; doi:10.1192/bjo.2022.74)
Supplement: Supplementary file 1 [file S2056472422000746sup001.docx]

**Supplemental Table S1.**

Overall sample characteristics (*N* = 228)

|  | Overall sample  (*N* = 228) | PTSD cohort  (*n* = 93) | Trauma cohort  (*n* = 135) | *t or x^2^* |
| --- | --- | --- | --- | --- |
| Demographics, *n (%)* or *M ± SD* |  |  |  |  |
| Age | 30.9 ± 6.0 | 30.8 ± 6.5 | 31.0 ± 5.6 | -0.243 |
| Pregnant as a teen (18-20) | 6 (2.6) | 4 (4.3) | 2 (1.5) | 1.709 |
| Low education (high school or less) | 41 (18.0) | 26 (28.0) | 15 (11.1) | 10.595^**^ |
| Poverty (<$15,000 household income) | 18 (7.9) | 12 (12.9) | 6 (4.4) | 5.418^*^ |
| High crime residence zip code | 8 (3.5) | 5 (5.4) | 3 (2.2) | 1.618 |
| Trauma History, *n (%)* |  |  |  |  |
| Childhood maltreatment | 78 (34.2) | 50 (53.8) | 28 (20.7) | 26.680^***^ |
| Adult abuse | 57 (25.0) | 36 (38.7) | 21 (15.6) | 15.745^***^ |
| Mental Health Profile, *n (%)* or *M ± SD* |  |  |  |  |
| Lifetime PTSD symptom count | 5.30 ± 4.96 | 10.60 ± 2.68 | 1.65 ± 1.94 | 27.567^***^ |
| B cluster sum | 1.31 ± 1.45 | 2.82 ± 1.14 | 0.27 ± 0.66 | 19.354^***^ |
| C cluster sum | 2.30 ± 2.23 | 4.60 ± 1.33 | 0.72 ± 1.02 | 23.782^***^ |
| D cluster sum | 1.69 ± 1.57 | 3.18 ± 1.02 | 0.66 ± 0.91 | 19.183^***^ |
| Dissociation score | 1.0 ± 2.08 | 1.70 ± 2.75 | 0.53 ± 1.26 | 3.844^***^ |
| Depersonalization/Derealization^ | 16 (7.0) | 15 (16.1) | 1 (0.7) | 19.984^***^ |
| Mental Health Treatment Use (*n*=140)^†^, *n (%)* |  |  |  |  |
| Past individual psychotherapy | 61 (43.6) | 31 (63.3) | 30 (33.0) | 11.892^**^ |
| Past psychiatric medication | 39 (27.9) | 28 (57.1) | 11 (12.1) | 32.171^***^ |
| Pregnancy individual psychotherapy | 11 (7.9) | 9 (18.4) | 2 (2.2) | 11.502^**^ |
| Pregnancy psychiatric medication | 9 (6.4) | 9 (18.4) | 0 (0.0) | 17.863^***^ |

Note. ^Coded using items c, e, f, h per the scoring instructions.

^†^One version of the survey included fewer items.

^*^ *p* < .05, ^**^ *p* < .01, ^***^ *p* < .001

**Supplemental Table S2.**

Significant interactions with childhood maltreatment to illustrate variations in dissociation (*N* = 228)

| SNP | Major Allele | Minor Allele | Protective  Allele or Genotype |  | Univariate ANOVA model | | |  | Childhood Maltreatment^a^  (1) | | |  | Protective Allele or Genotype  (2) | | |  | (1) x (2) | | |
| --- | --- | --- | --- | --- | --- | --- | --- | --- | --- | --- | --- | --- | --- | --- | --- | --- | --- | --- | --- |
|  |  |  |  |  | *F* | *ε^2^* | *p* |  | *F* | *ε^2^* | *p* |  | *F* | *ε^2^* | *p* |  | *F* | *ε^2^* | *p* |
| rs2254298^b^ | G | A | G |  | 9.85 | 0.12 | **0.000004** |  | 18.37 | 0.08 | **0.000027** |  | 8.87 | 0.04 | **0.0032** |  | 14.07 | 0.06 | **0.00022** |
| rs237897 | C | T | C/C |  | 2.66 | 0.11 | 0.023 |  | 3.80 | 0.03 | 0.024 |  | 0.75 | 0.00 | 0.388 |  | 3.23 | 0.03 | 0.041 |

Note. Results were not corrected for multiple testing, and instead the adjusted p-value=0.0089 was used as the threshold for significance.

^a^ Childhood maltreatment history variable has 3 categories: none (0), 1, and 2 or more types of childhood maltreatment.

^b^ No one showed ‘1’ childhood maltreatment history among European Americans with the minor allele homozygote (A/A) of rs2254298; thus, childhood maltreatment history variable has 2 categories only for this SNP: none (0) vs. 1 or more types of childhood maltreatment.
